# Atherosclerosis Calcification: Focus on Lipoproteins

**DOI:** 10.3390/metabo13030457

**Published:** 2023-03-21

**Authors:** Jaap G. Neels, Georges Leftheriotis, Giulia Chinetti

**Affiliations:** 1 Université Côte d’Azur, INSERM, C3M, 06200 Nice, France; jaap.neels@univ-cotedazur.fr; 2 Université Côte d’Azur, CHU, CNRS, LP2M, 06000 Nice, France; georges.leftheriotis@univ-cotedazur.fr; 3 Université Côte d’Azur, CHU, INSERM, C3M, 06200 Nice, France

**Keywords:** vascular calcification, lipids, inflammation, cardiovascular disease, cholesterol, calcium

## Abstract

Atherosclerosis is a chronic inflammatory disease characterized by the accumulation of lipids in the vessel wall, leading to the formation of an atheroma and eventually to the development of vascular calcification (VC). Lipoproteins play a central role in the development of atherosclerosis and VC. Both low- and very low-density lipoproteins (LDL and VLDL) and lipoprotein (a) (Lp(a)) stimulate, while high-density lipoproteins (HDL) reduce VC. Apolipoproteins, the protein component of lipoproteins, influence the development of VC in multiple ways. Apolipoprotein AI (apoAI), the main protein component of HDL, has anti-calcific properties, while apoB and apoCIII, the main protein components of LDL and VLDL, respectively, promote VC. The role of lipoproteins in VC is also related to their metabolism and modifications. Oxidized LDL (OxLDL) are more pro-calcific than native LDL. Oxidation also converts HDL from anti- to pro-calcific. Additionally, enzymes such as autotaxin (ATX) and proprotein convertase subtilisin/kexin type 9 (PCSK9), involved in lipoprotein metabolism, have a stimulatory role in VC. In summary, a better understanding of the mechanisms by which lipoproteins and apolipoproteins contribute to VC will be crucial in the development of effective preventive and therapeutic strategies for VC and its associated cardiovascular disease.

## 1. Introduction

According to the World Health Organization, atherosclerosis represents the main cause of mortality, leading to cardiovascular and vascular diseases (including myocardial infarction, stroke, peripheral arterial diseases, cerebrovascular diseases) [1,2]. Thus, atherosclerosis represents a major public health problem. Common atherosclerosis risk factors are age, hypertension, smoking, sedentarity, dyslipidemia, obesity and type 2 diabetes [3]. Atherosclerosis is a chronic inflammatory process due to the formation of plaques that build up inside the large and median arteries (aorta, carotids, femoral arteries) as a result of the deposition of fat, cholesterol, calcium, fibrotic tissue, cells and cellular debris [4]. Over decades, a plaque hardens and narrows the arteries, leading to clinical manifestations [2]. The development of atherosclerosis involves the activation of various cell types (including endothelial cells, smooth muscle cells (SMC), lymphocytes, monocytes and macrophages) in the intima of the arteries, which results in a local inflammatory response [4]. An increase in circulating LDL (low density lipoprotein)-cholesterol levels and the subsequent accumulation of oxidized LDL (OxLDL) in the subendothelial space triggers the recruitment and retention of monocytes and lymphocytes in the arterial wall. In the intima, monocytes differentiate into macrophages, which scavenge lipoprotein particles, accumulate lipids (mainly cholesterol) and become foam cells [5]. These macrophage-derived foam cells secrete inflammatory molecules and factors that further promote lipoprotein retention, affect SMC phenotype, proliferation and migration to the intima, degrade the extracellular matrix and sustain inflammation [4]. While lesional macrophages are mainly derived from blood monocytes, it has been shown that lesional macrophage-like cells can also be derived from SMC [6]. Moreover, it has been also reported that tissue-resident macrophages can renew themselves by local proliferation [7]. Progression of atherosclerosis is characterized by apoptosis of these resident macrophages in the lipid core of the lesion. The clearance of apoptotic cells is mediated by phagocytes, mostly macrophages, which recognize and internalize dead cells in a process termed efferocytosis [8,9]. In early lesions, phagocytes readily clear apoptotic cells, avoiding further progression of atherosclerosis. In chronic, advanced lesions, however, efferocytosis is no longer sufficient to engulf all dead cells, and the gradual accumulation of apoptotic debris results in the formation of a necrotic core, which triggers further inflammation, necrosis and thrombosis [8,9]. Plaque necrosis associated to fibrous cap thinning provokes plaque rupture, resulting in acute luminal thrombosis leading to atherosclerosis clinical manifestations [10]. In addition to drug administration, such as anti-platelets and statins [11], cardiovascular interventions such as angioplasty and stenting play a central role in the treatment of atherosclerotic-associated diseases, although restenosis remains a risk limiting factor for these procedures [12]. 

An important element in the atherosclerotic process development is vascular calcification, defined as an inappropriate deposition of calcium minerals in arterial wall beds. In fact, atherosclerotic plaque stability depends on the differential amounts, sizes, shapes and location of calcification [13].

The purpose of this updated review of the literature is to present the current state of knowledge on the role of lipoproteins in vascular calcification by discussing clinical and experimental studies.

## 2. Vascular Calcification Process

Vascular calcification (VC) is a complex process by which calcium deposits accumulate within the vessel walls and valves, resulting in the formation of extra-cellular calcified nodules. It is a gradual process that occurs over time and is associated with aging and acquired chronic metabolic diseases such as diabetes or chronic kidney disease [14]. 

Although vascular and valvular calcifications share risk factors and molecular pathways, several physio-pathological key differences between these two forms of VC are important to consider [15]. One of the main differences is linked to the histological structure of calcification sites, with valves consisting of tri-layered structures defined as fibrosa (connective tissue providing strength), spongiosa (mucopolysaccharides facilitating movement), and ventricularis (elastin contributing to flexibility), while vessel walls are constituted mainly by vascular smooth muscle cell (VSMCs) and elastin-rich and connective layers. Furthermore, cells involved in VC also differ, with VSMCs in vessels versus interstitial cells (VICs) in valves. Differences in how vessels and valves are exposed to shear stress also impacts VC, and vessels calcify faster than valves.

VC within vessels can be classified into two types: intimal and medial [16]. While intimal and medial VC are two distinct types of VC that have different origins and underlying mechanisms, both can lead to cardiovascular complications. Intimal VC occurs within the intima of the vessel wall and is associated with inflammation and atherosclerosis [17]. This type of VC is initiated by the accumulation of lipids and other pro-inflammatory molecules in the intima of the vessel, leading to the formation of an atheroma. As the atheroma progresses, calcified nodules often form in the advanced stages of atherosclerosis and can be observed in the aorta, coronary arteries and carotids. Medial VC, on the other hand, occurs within the media of the vessel wall, and is often observed in diabetic or chronic kidney disease (CKD) patients [18]. Medial VC is initiated by the loss of elastic fibers and proteoglycans in the media of the vessel wall, leading to structural changes and eventually the formation of calcium deposits that occur in parallel or independently of atherosclerosis, and can often be observed in femoral, tibial and uterine arteries [19]. Medial VC leads to a reduction in the elasticity of the vessel wall (arterial stiffness), which in turn induces systolic hypertension through impaired cardiovascular hemodynamics, subsequently resulting in cardiovascular disease (CVD) [19]. Interestingly, the pathogenic role of isolated medial calcification is unclear and may be mostly linked to its association to occlusive arterial lesions [20].

Contrary to the initial thoughts of a degenerative process, the mechanism of VC is influenced by a wide range of systemic factors, including aging, diabetes, CKD and cardiovascular risk factors such as hypertension, dyslipidemia and smoking [21]. Several key molecular mechanisms have been identified to contribute to VC, including inflammation, oxidative stress and mineral metabolism disorders, mainly hyperphosphatemia and vitamin D deficiency [22]. Moreover, genetic polymorphisms have been identified to contribute to some types of VC [23].

Atherosclerotic plaque stability is also closely linked to the size and location of VC within the plaque. Macrocalcifications, which are large (>0.5 mm) and visible calcifications, are typically associated with increased plaque stability, while microcalcifications, which are small (≤0.5 mm) and almost undetectable by imaging techniques, are linked to plaque rupture [24]. Macrocalcifications are often found in the deeper layers of the plaque, away from the lumen of the vessel. These calcifications are less likely to disrupt the mechanical properties of the plaque, and are therefore considered as a stabilizing effect that limits plaque rupture. Additionally, macrocalcifications tend to be surrounded by a fibrous cap, which provides additional structural support to the plaque and further increases its stability. On the other hand, microcalcifications are often found in the thin fibrous cap of the plaque, near the lumen of the vessel; these calcifications are more likely to favor possible plaque rupture. Additionally, microcalcifications are often found in association with high-risk plaque features such as a large lipid core and high macrophage infiltration. However, only a small subset of microcalcified plaques has the potential for rupture [25].

The cellular and molecular mechanisms underlying VC are complex and multifactorial; among them, the roles of VSMCs and VICs have been extensively studied. In response to various pro-osteogenic signals, such as high glucose, inflammation and oxidative stress, VSMCs and VICs can undergo a process of trans-differentiation, in which they acquire an osteoblastic phenotype and begin to deposit calcium and other minerals within the vessel wall or valve, respectively [26]. This process is mediated by the expression of osteogenic transcription factors such as RUNX2, which promote the differentiation of VSMCs and VICs into osteoblasts [27]. Additionally, VSMCs/VICs also express enzymes such as alkaline phosphatase (ALP), which converts pyrophosphate into phosphate, an important factor for the nucleation of hydroxyapatite crystals.

Macrophages, on the other hand, play a key role in the regulation of VSMC/VIC-mediated VC [28]. Macrophages can differentiate into different subpopulations, such as pro-inflammatory or anti-inflammatory, depending on their microenvironmental signals. Pro-inflammatory macrophages can release inflammatory molecules that can activate VSMCs/VICs and promote their trans-differentiation into osteoblasts, while anti-inflammatory macrophages can release anti-inflammatory and anti-osteogenic molecules inhibiting the trans-differentiation of VSMCs/VICs [29]. Therefore, the balance between pro- and anti-inflammatory macrophages in the vessel wall strongly influence the development of VC.

Overall, VC is a complex process mediated by a variety of cellular and molecular mechanisms, influenced by a wide range of systemic factors. A better understanding of these mechanisms and factors will be crucial in the development of effective preventive and therapeutic strategies for VC and its associated cardiovascular disease. 

## 3. Lipoproteins and Vascular Calcification

### 3.1. Lipoproteins and Their Subfractions: Results from Clinical Studies

Clinical studies linking lipoprotein subfractions to VC have been performed in different patient populations by several research groups. Type 1 diabetic patients without a history of CVD show lower levels of circulating osteocalcin positive (OCN+) monocytes, considered as osteogenic precursor cells of myeloid origin, compared to subjects with CVD [30]. Interestingly, the concentration of OCN+ monocytes inversely correlated with total high-density lipoprotein (HDL) cholesterol levels, as well as with large and intermediate HDL-subfractions, but not with small HDL [30]. The amount of OCN+ monocytes was not related to total cholesterol, LDL cholesterol, nor triglycerides. However, the analysis of LDL subfractions showed a trend towards a positive association with small and dense LDL. The use of lipid lowering drugs was not associated with the number of OCN+ cells. The large HDL subfraction was strongly inversely correlated with coronary artery calcification (CAC) in healthy postmenopausal women [31]. Moreover, small, medium and large very low-density lipoprotein (VLDL) subfractions all positively correlated with CAC. The concentration of small dense LDL positively correlated with CAC, which was not the case for medium and large LDL. Furthermore, the association between small dense LDL and intracranial arterial calcification, which increases the risk of ischemic stroke and cognitive decline [32], was evaluated. Serum small dense LDL levels correlated with the hospital admission NIHSS (National Institutes of Health Stroke Scale) score, reflecting the severity of acute cerebral infarctions. The average concentration of small dense LDL was higher in patients who died during hospitalization compared to patients who survived [32].

The number of circulating LDL particles (LDL-P) represents an alternative measure of LDL concentration, allowing a better understanding and measure of residual CVD risk in patients achieving the recommended LDL-cholesterol concentrations upon statin treatment. Indeed, LDL-P has been shown to be a better CVD risk predictor than LDL-cholesterol [33]. In intermediate coronary artery disease (CAD) risk factor subjects (aged 40 to 69 years, 67.6% male), in the absence of treatment with statin or niacin, the LDL-P showed a stronger association with CAC than the traditional lipoprotein concentration [34]. Patients with the highest tercile of total LDL-P had an approximately 3.7-times higher risk to develop CAC than those with the lowest tercile. Similar results were obtained in a population of Japanese men (aged 40 to 79 years), where LDL-P were significantly associated with the CAC, independently of LDL cholesterol [35].

A study conducted in a sub-population of the Multi-Ethnic Study of Atherosclerosis (MeSA) cohort, without subclinical atherosclerotic CVD and without lipid lowering treatment, showed that concentrations of apolipoprotein B (apoB) were associated with CAC in patients older than 45 years [36]. However, this study provided only modest additional value of apoB for CAC prevalence, incidence or progression beyond the measure of LDL cholesterol and non-HDL cholesterol. Moreover, it has been reported that the LDL cholesterol/apoB ratio, representing the predominance of small dense LDL, was better compared to the apoB alone in the diagnosis of CAC [37].

By analyzing the same MeSA cohort, the concentration of HDL particles (HDL-P), representing the sum of HDL subclass particles, considered as a novel marker that inversely associated with CVD risk, was evaluated [38]. Results showed that the high HDL-P concentrations were associated with lower odds of CAC presence and progression, which is in line with the inverse association between HDL-P and cardiovascular risk [39].

Interestingly, results from the ATLANTA I study, analyzing the relationship between lipoproteins and plaque components by computed tomography angiography (CTA) and intravascular ultrasound (IVUS), showed that apoB-containing lipoproteins, as well as HDL-P, were involved [40]. Indeed, apoB particles were associated with a higher proportion of non-calcified plaque and a lower proportion of calcified plaques. Concerning HDL-P, small HDL were also associated with larger plaque burden and more non-calcified plaques, whereas larger HDL and pre-*β*2 HDL were associated with less calcification and less stenosis, but a higher proportion of fibrotic tissue. Moreover, small lipid-poor HDL (pre-*α*4, pre-*α*3 and *α*3 HDL) were associated with a lower proportion of calcified and a higher proportion of non-calcified plaques.

Analysis of the relative importance of non-HDL cholesterol concentration on CAC at different stages of life has been evaluated, including adolescence (12–18 years), young adulthood (21–30 years) and midadulthood (33–45 years) [41]. Results showed that elevated non-HDL cholesterol at each life stage was associated with CAC in mid-adulthood. Interestingly, non-HDL cholesterol in adolescence showed the strongest association with the presence of CAC in adulthood.

The atherogenic index of plasma (AIP), calculated as the log of the triglycerides/HDL cholesterol ratio, has been suggested to be more closely related to CVD risk, compared to individual lipoprotein cholesterol concentration [42]. The association between AIP and CAC progression has been studied in asymptomatic Korean adult subjects [43]. Results showed that the presence of CAC at baseline, and its progression during a 3.3 year follow-up, were more frequently observed in patients with the higher AIP quartile. However, the AIP was associated with the risk of CAC progression over the traditional CVD risk factors in subjects without heavy CAC at the baseline [43].

Some studies have also looked at the direct involvement of individual apolipoproteins in the process of human aortic valve calcification. The concentration of apoAI, the major component of HDL, was higher in control than in human stenotic aortic valves. In these tissues, apoAI surrounded calcium depots and colocalized with apoB, apoE and osteoprotegerin (OPG), a calcification inhibitor [44]. Moreover, apo(a) was prominent in aortic valves with calcified nodules or large calcification areas [45]. Indeed, by comparing non-fibrotic/non-calcified to fibrotic/calcified aortic valve tissues, the authors identified the presence of apoCIII, apoB, and to a lesser extent apoJ and apoE, which was more abundant around calcified regions. In particular, apoCIII was detected in both lipid-rich and lipid-poor areas surrounding calcified nodules, suggesting that apoCIII may contribute to calcification independently of its role in lipoprotein metabolism [46]. These results suggest that apolipoproteins may play critical roles in calcification initiation and progression, either through direct pathological interaction with cells and the extra-cellular matrix, or through their functions as lipid carriers. However, patients with aortic valve sclerosis exhibited higher concentrations of serum apoCII, apoCIII and apoCIII contained in VLDL + LDL fractions [47]. Heterozygous carriers of a null mutation (R19X) in the gene encoding apoCIII, compared with noncarriers, had lower fasting and postprandial serum triglycerides, higher levels of HDL-cholesterol, lower levels of LDL-cholesterol and less coronary artery calcification [48,49].

The blood determination of the basic fractions of the lipid profile (total cholesterol, LDL-C, HDL-C and triglycerides) gives only basic knowledge about the patient’s lipid status. Moreover, lipoproteins can also undergo modifications (oxidation, nitration, glycation, alkylation, aggregation, etc.), especially under oxidative stress [50], which can lead to the formation of more atherogenic lipoproteins that are not routinely measured in clinical practice. 

Interestingly, circulating concentrations of oxidized HDL (OxHDL), which are characterized by reduced anti-inflammatory properties compared to normal HDL, were significantly higher in patients with severe calcific aortic valve disease (CAVD), compared to age and gender-matched subjects without CAVD [51]. Indeed, the decrease in OxHDL concentration was associated with an attenuation of the CAC progression in hypercholesterolemic patients under pitavastatin treatment [52].

Measurement of lectin-like oxidized low-density lipoprotein (LDL) receptor-1 (LOX-1) ligand containing apoAI (LAA), an indicator of modified HDL that presents impaired anti-atherogenic functions [53], showed that LAA was associated with CAC, independently of the HDL cholesterol and particle concentrations in middle aged (<65 years) Japanese men [54].

Patients with heterozygous or homozygous mutations of the LDL receptor, characterized by the familial hypercholesterolemia (HeFH) showed increased prevalence of aortic valve calcification, compared with control subjects [55]. Moreover, the progression of the aortic calcification was followed over a period of >8 years in HeFH patients [56]. Aortic calcification increased in all patients in an exponential fashion with respect to age, which remains the most important factor that affects the rate of aortic calcification [56]. The calcification process continued independently of total cholesterol or LDL-C levels. Indeed, age and *LDLR*-negative mutations were strong predictors of aortic valve calcification. 

Altogether, these results suggest that analysis of lipoprotein sub-fractions may improve the prediction of CAD in patients beyond the conventional lipid parameters and risk factors. 

#### 3.1.1. Lipoprotein (a) Lp(a)

**Figure 1:**
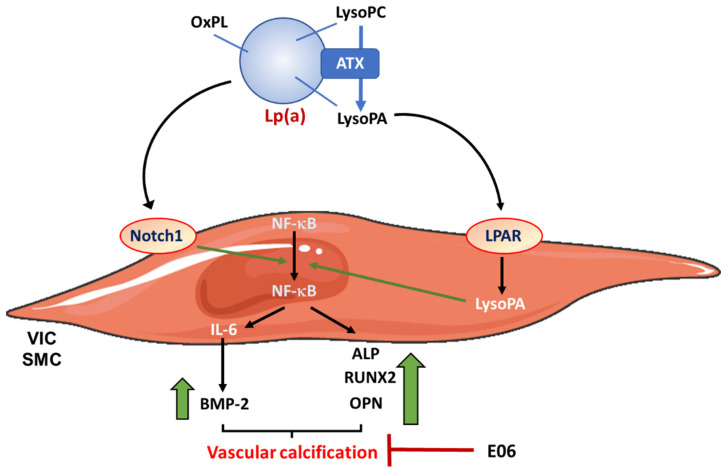
Lp(a) in vascular calcification: mechanisms of actions. Lp(a) is a lipoprotein transporting oxidized phospholipids (OxPL), as well as autotaxin (ATX), a lysophospholipase D that converts lysophosphatidylcholine (LysoPC) from OxPL into lysophosphatidic acid (LysoPA). Lp(a) increases cell mineralization, as well as the expression of pro-calcific proteins, by a mechanism involving the activation of the Notch1 signaling pathway, which, in turn, allows nuclear translocation of the nuclear factor-κB (NF-κB). This results in the induction of IL-6 expression, increasing BMP-2 concentrations, as well as the induction of the expression of RUNX2, osteopontin (OPN) and ALP. LysoPA also increases NF-κB nuclear translocation. These effects are blocked by the presence of E06, a natural monoclonal antibody against OxPL. LPAR: lysophosphatidic acid receptor.

Lipoprotein (a) (Lp(a)), an LDL-like particle, characterized by the presence of the apo(a) component that is covalently linked to the apoB moiety by the disulfide bound [57], is considered as a strong marker for cardiovascular disease. Lp(a) is the only apoB-containing lipoprotein that transports oxidized phospholipids (OxPL) [58]. Lp(a) also carries autotaxin (ATX), a lysophospholipase D enzyme that converts lysophosphatidylcholine (LysoPC) from OxPL into lysophosphatidic acid (LysoPA) (Figure 1) [45]. The Lp(a) plasma concentration is 90% determined by genetics [59]. The role of Lp(a) has been largely studied in VC, particularly at the aortic valve level. Indeed, an elevated Lp(a) concentration has been associated with approximately one-third of aortic stenosis cases [60]. Calcified aortic valves expressed OxPL epitopes and ATX, as well as apo(a) [45]. Interestingly, ATX expression and activity were higher in mineralized aortic valves compared to control non-mineralized tissues [61]. Moreover, analysis of calcified aortic valves reveals that tissue ATX is probably transported from blood by Lp(a), but also can be directly secreted by VICs [61]. Lp(a) concentration independently correlated with the presence and the severity of CAC in a study enrolling 2806 patients [62].

Patients with the higher Lp(a) tertile had higher valve calcification, as well as higher progression of valvular calcium score, compared to those with the lower tertiles [63]. Elevated Lp(a) and OxPL levels were associated with prevalent calcific aortic valve stenosis in patients. In individuals with elevated Lp(a), evidence of aortic valve microcalcification by 18F-sodium fluoride positron emission tomography/computed tomography was present before the development of clinically manifested calcific aortic valve stenosis, suggesting a role for Lp(a) in the development of the disease [64].

Analysis of the European Prospective Investigation into Cancer–Norfolk cohort demonstrated that individuals with the highest Lp(a) tertile had a 57% higher risk of aortic valves stenosis, and that the rs10455872 variant of the LPA gene was associated with the higher Lp(a) concentrations [65,66]. In line, in a secondary analysis of the “Cardiovascular Outcomes Research with PCSK9 Inhibition in Subjects with Elevated Risk (FOURIER)” [67], increasing Lp(a) concentrations were associated with a higher risk of aortic valve stenosis, including progression or need for valve replacement. Lp(a) measured in routine clinical care over a 14-year follow-up period was higher in subjects with calcified aortic valve stenosis, independent of their sex [68]. Moreover, analysis of asymptomatic HeFH patients showed that 38.2% of them present aortic valve calcification, and that Lp(a) remained a significant predictor of valve calcification after adjustment for all significant covariables [69]. Analysis of the ASTRONOMER cohort (Aortic Stenosis Progression Observation: Measuring Effects of Rosuvastatin) demonstrated that elevated levels of Lp(a)-apoCIII complexes were detected in patients with pre-existing mild-moderate calcific aortic stenosis, and who display rapid progression of the pathology [49].

Analysis of two Dutch cohorts of asymptomatic subjects (the Rotterdam and the Amsterdam studies) revealed that higher Lp(a) concentrations were independently associated with the presence of aortic valve calcification in both cohorts. In patients with aortic valve calcification, Lp(a) correlated with increased calcific burden. Aortic valve calcification was already highly prevalent in younger individuals with Lp(a) above the 80th percentile, emphasizing the need for early identification of these subjects [70]. Moreover, very recently it has been reported, by analyzing the Rotterdam study on the apparently healthy general population, that Lp(a) levels were associated with aortic valve calcification onset, but not with its progression in subjects with an already established pathology on a 14-year follow-up [71]. This suggests that Lp(a) lowering strategies may be most effective in the early stage of calcification. These results can appear contradictory with some other previously published results, suggesting that Lp(a) drives valve calcification and disease progression [63,72], but can be explained by difference in population selection (established calcification vs. apparent healthy status), duration of the follow-up period, and choice of the final endpoint.

Finally, concerning the link between ATX and calcific aortic valve stenosis, it has been reported that ATX mass and activity were independently associated with the pathology compared to patients with coronary artery disease without aortic valve disease [73]. Indeed, patients with both higher ATX activity and Lp(a) or OxPL-apoB had an elevated risk of calcific aortic valve stenosis. 

#### 3.1.2. Proprotein Convertase Subtilisin/Kexin Type 9 (PCSK9)

Proprotein convertase subtilisin/kexin type 9 (PCSK9) is a hepatic enzyme stimulating LDL-R degradation, thus resulting in increased circulating LDL concentrations [74]. The use of specific PCSK9 inhibitors, particularly monoclonal antibodies, such as alirocumab and evolocumab, represents a novel promising approach to reduce circulating LDL-cholesterol.

The role of PCSK9 in calcification, notably in aortic valve calcification (AVC), is well established. PCSK9 is highly expressed in mouse [74] and human calcified aortic valves, particularly in VICs [74,75]. Indeed, old PCSK9-deficient mice presented lower AVC than the controls, and mouse PCSK9-deficient VICs are partially protected from calcification in vitro [74]. Patients with the PCSK9 loss of function mutation (PCSK9 R46L), characterized by decreased circulating Lp(a) and LDL-cholesterol, had reduced risk of calcific aortic stenosis [76]. Exploratory investigation of the randomized clinical trial “FOURIER” showed that patients under evolocumab treatment, on top of statin administration, had a 50% decrease in the incidence of calcification during a follow-up period of approximatively 2.2 years [67].

**Figure 2:**
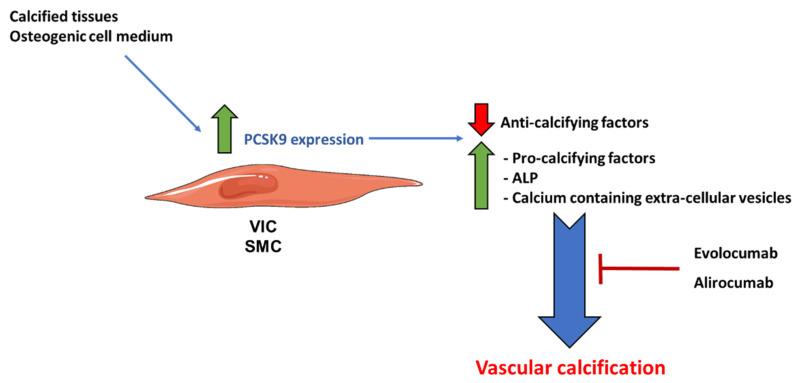
Role of PCSK9 in vascular calcification. PCSK9 expression is high in mouse and human calcified aortic valves, as well as in vitro VICs in the presence of pro-osteogenic medium. PCSK9 increases the number of secreted extracellular vesicles, containing more calcium and ALP, enhances the expression of pro-calcifying markers and lowers those of anti-calcifying mediators. These effects are blocked by evolocumab and alirocumab, two specific antibodies against PCSK9. ALP: alkaline phosphatase.

Treatment with alirocumab plus statin, compared to standard statin therapy, significantly decreased LDL cholesterol in both groups, while the absolute reduction of LDL cholesterol levels was higher in patients treated with alirocumab. Additionally, patients in the alirocumab group demonstrated a significant reduction of Lp(a) levels, not observed under the standard statin treatment. CAC progression was significantly lower in the alirocumab group than in the standard statin group [77]. Altogether, these results suggest the potential beneficial contribution of PCSK9 inhibition to VC (Figure 2).

### 3.2. Lipoproteins and Extra-Cellular Matrix Mineralization: Results from Experimental Studies

Several experimental studies have investigated the cellular and molecular mechanisms underlying the relationship between lipoproteins and calcification.

#### 3.2.1. Lipoproteins

At late atherosclerosis stages, HDL plays a role in the prevention of VC by inhibiting the trans-differentiation of VSMCs. HDL also reduced the activity of ALP, a marker of osteogenic differentiation of osteoblastic cells [78], and inhibited IL-1β, IL-6 and minimally oxidized LDL-induced osteogenic activity [79]. Interestingly, these effects were mimicked by the lipid moiety of HDL, but not by the HDL-associated apolipoproteins or reconstituted HDL. 

Moreover, in vitro addition of HDL to human THP-1 and U937 monocytic cell lines significantly decreased the number of OCN+ monocytes induced by OxLDL, via a mechanism involving the HDL receptor SR-B1 [30]. However, non-oxidized LDL had no effect on the expression of OCN and did not interact with HDL. This represents a novel mechanism by which HDL protects against cardiovascular disease by counteracting monocyte differentiation into pro-calcific cells. However, HDL are prone to oxidative modifications, leading to changes from protective to pro-atherogenic and pro-inflammatory properties. Indeed, OxHDL enhanced cellular osteogenic activity [79]. More precisely, OxHDL enhanced vascular cell mineralization by increasing ALP activity, as well as by inducing the expression of osteogenic factors (RUNX2, BMP-2, WNT5a, Osterix, etc.) [51,80].

Enzyme-modified non-oxidized LDL (ELDL), which have been detected in human calcific aortic valve disease [81], represent one of the many forms of modified LDL. This LDL modification occurs through the action of hydrolytic enzymes and differs from “classical” OxLDL since they lack oxidized lipids. Treatment of cultured human coronary artery SMC with ELDL in a phosphate-containing medium promoted VC by inhibiting the expression of calcification inhibitors such as matrix gla protein and ENPP-1. The latter converts extracellular ATP to adenosine and generates pyrophosphate, an inhibitor of calcification. Up-regulated expression of genes promoting calcification (RUNX2, ALP, BMP-2, Osterix…) was also observed [82]. 

Other lipoproteins including OxLDL and Lp(a) have been shown to activate innate immune responses in cells leading to a gain of pro-calcific phenotypes in calcific aortic valve disease. OxLDL induced the expression of the inorganic phosphate transporter Pit-1 and of BMP-2 in primary human VICs [83]. Moreover, OxLDL increased the RANKL expression in human SMC, without affecting the RANKL decoy receptor OPG [84]. Interestingly, the lipid extracts of these OxLDL reproduced the effects of the whole particle. Moreover, OxLDL-derived LysoPA promoted mineralization and osteogenic transition of human VICs. Addition of Ki16425, an inhibitor of LysoPA receptor 1 (LPAR1/Edg-2) and LPAR3/Edg-7, to cultured VIC prevented OxLDL-induced mineralization, suggesting that LysoPA produced by OxLDL promoted VIC mineralization [85]. 

Cholesterol per se has also been reported to control calcification [86]. Indeed, murine aortic SMCs from LDL-R deficient (LDLR-/-) mice, cultured under pro-calcifying conditions, displayed less intracellular cholesterol, and are characterized by lower ALP activity and matrix calcium deposition compared to SMCs isolated from control mice. Treatment of cells from control mice, with lipoprotein deficient serum (LPDS), resulted in a reduced matrix calcium deposition, compared to the normal serum. Interestingly, these effects were rescued by addition of cell permeable cholesterol. Finally, treatment of cells from LDLR-/- mice with mevastatin, to reduce intracellular cholesterol synthesis, and with forskolin, a PKA activator known to promote cell mineralization, resulted in a significant reduction of the matrix calcium deposition. Moreover, treatment of SMC with 25-hydroxy cholesterol upregulated ALP expression, thus increasing calcification [87]. Finally, reduction of circulating cholesterol concentration in ApoE-deficient mice led to reduced aortic root calcification [88]. Altogether, these results suggest that lipoprotein and cholesterol metabolism is involved in extra-cellular mineralization (Table 1).

#### 3.2.2. Apolipoproteins

Concerning the role of apolipoproteins, it has been reported that treatment of primary human VICs with human apoCIII, in the presence of a pro-calcifying medium, led to a significant increase of calcium deposition by a mechanism involving mitochondrial dysfunction and inflammatory pathways [46]. In the same experiments, addition of apoAI significantly reduced the VIC calcification, thus supporting its protective role. This has been confirmed in vivo in animal studies, where injection of apoAI mimetic peptides significantly reduced calcification both in mice and rabbits [89,90,91]. Finally, treatment of human valve myofibroblasts with either apoAI, HDL_2_ or HDL_3_ increased the secretion of OPG, while exerting anti-inflammatory actions and repressing expression of TNFα [44] (Table 1).

**Table 1:** Mechanisms of action of lipoproteins and apolipoproteins in vascular calcification.

Apo and Lipoproteins	Cell or Animal Models	Mechanisms of Action	References
**HDL**	Bovine VSMCs	Inhibit VSMCs trans-differentiation by reducing ALP activity. Inhibit IL-1β, IL-6 secretion and minimally OxLDL-induced osteogenic activity.	[79]
	Human THP-1 and U937 monocytic cell lines	Decrease the number of OCN+ monocytes induced by OxLDL by a mechanism involving the SR-B1 receptor.	[30]
**OxHDL**	Bovine VSMCs	Enhance vascular cell mineralization by increasing ALP activity.	[79]
	Human VSMCs and VICs	Induce the expression of osteogenic factors (RUNX2, BMP-2, WNT5a, Osterix, etc.).	[51,80]
**ELDL**	Human coronary artery SMCs	Inhibit the expression of calcification inhibitors such as matrix gla protein and ENPP-1. Up-regulate the expression of genes promoting calcification (RUNX2, ALP, BMP, Osterix…).	[82]
**OxLDL**	Human VICs	Induce the expression of the inorganic phosphate transporter Pit-1 and of BMP-2.	[83]
	Human SMCs	OxLDL-derived LysoPA promotes mineralization and cellular osteogenic transition.	[84]
		Increased the RANKL expression in human SMC, without affecting the RANKL decoy receptor osteoprotegerin (OPG). The lipid extracts of OxLDL reproduce the effects of the whole particle.	[84]
**Cholesterol**	Aortic SMCs from LDL-R deficient (LDLR-/-) mice, cultured under pro-calcifying conditions	Lower ALP activity and matrix calcium deposition compared to SMCs isolated from control mice.	[86]
	Cells from control mice	Treatment with lipoprotein deficient serum (LPDS), reduces matrix calcium deposition, compared to the normal serum.	[86]
	Cells from LDLR-/- mice	Mevastatin reduces the matrix calcium deposition.	[86]
	Mouse SMCs	25-hydroxy cholesterol upregulates ALP expression and increases calcification.	[87]
	Reduction of circulating cholesterol concentration in ApoE-deficient mice	Reduces aortic root calcification.	[88]
**apoCIII**	Human VICs	Increases calcium deposition by a mechanism involving mitochondrial dysfunction and inflammatory pathways.	[46]
**apoAI**	Human VICs	Reduces calcification.	[46]
	Mice and rabbits	Mimetic peptides significantly reduced calcification.	[89,90,91]
** apoAI, HDL_2_, or HDL_3_**	Human valve myofibroblasts	Increase OPG secretion.	[44]

Summary of the main actions of lipoproteins, apolipoproteins and cholesterol in vascular calcification, in cells and animal models. OCN: osteocalcin; OxHDL: oxidized HDL; ELDL: enzyme-modified LDL; OxLDL: oxidized LDL; apo: apolipoprotein; SR-B1: scavenger receptor B1; OPG: osteoprotegerin; ENPP-1: ectonucleotide pyrophosphatase/phosphodiesterase 1; VICs: valvular interstitial cells; SMCs: smooth muscle cells.

#### 3.2.3. Lp(a) and PCSK9

Mechanisms by which Lp(a) controls VIC and SMC calcification have been determined in vitro [63]. Regarding the understanding of the mechanisms of Lp(a)-mediated calcification, in vitro results showed that treatment of human aortic SMC with native Lp(a) increased cell mineralization, as well as the expression of pro-calcific proteins, by a mechanism involving the activation of the Notch1 signaling pathway, which, in turn, allows translocation of the nuclear factor-κB (NF-κB) [62]. Indeed, NF-κB silencing reduced Lp(a)-induced mineralization [62]. Moreover, Lp(a) stimulated the release of extracellular vesicles able to calcify collagen matrix, independent of the presence of cells [92]. Native Lp(a) increased expression of BMP-2, OPN and RUNX2, an effect attenuated by pre-incubation of Lp(a) with a natural monoclonal antibody against the OxPL (E06). These data have been confirmed using a specific construct with defective binding of OxPL, thus indicating that the OxPL moiety of Lp(a) was responsible for its effects on calcification (Figure 1). Interestingly, the involvement of OxPL has been also confirmed in vivo in LDLR-deficient mice expressing a fragment of the E06 antibody [93]. Indeed, the presence of E06 decreased aortic valve calcium content by approximately 41%. 

The role of ATX, as well as LysoPC and LysoPA, on cell mineralization has also been studied. Treatment of human VICs with both LysoPC and LysoPA, in the presence of calcifying medium, significantly increased cell mineralization through a NF-κB/IL-6/BMP2 pathway [61]. The pro-mineralizing effects of LysoPC were abrogated in the presence of ATX siRNA. In vivo, treatment with LysoPA increased the calcium deposition in aortic valve leaflets in a mouse model [61] (Figure 1). 

Mechanistically speaking, PCSK9 mRNA and secreted protein increased in VICs exposed to a pro-osteogenic medium [75]. Human and rat SMC overexpressing PCSK9 had an increased mineralization, released a higher number of extracellular vesicles, containing more calcium and ALP, and expressed more pro-calcifying markers and lower anti-calcifying mediators than control cells [94]. VIC calcification positively correlated with the amount of secreted PCSK9 [75] (Figure 2). An important issue regarding the pro-calcifying effects of PCSK9 is whether they were associated with intra- or extra-cellular protein. Indeed, while neither the addition of extracellular recombinant PCSK9 nor treatment with evolocumab to PCSK9-over-expressing SMC had any effect on cell calcification [94], another study reported that addition of a PCSK9 neutralizing antibody significantly reduced calcium accumulation in human primary VICs [75]. These discrepancies could be explained by differences in the cell model used or can be due to differences in the composition of osteogenic medium.

## 4. Lipid-Lowering Drugs in VC and AVC: The Good and the Bad

Among the lipid-lowering drugs, statins represent the most prescribed class of drugs globally. Since 1990, a large amount of literature has acknowledged the fact that statins are associated with a beneficial effect on atherosclerotic plaques by slowing progression of coronary atherosclerosis [95]. In addition to the reduction of cholesterol levels, the beneficial effect of statins on plaque regression results from complex pleiotropic effects, including local anti-inflammatory effects, changes in phenotypic plaque composition and the reduction of high-risk plaques [96]. Paradoxically, statins have been reported to increase VC and AVC [95,97,98]. This calcifying effect is considered beneficial by favoring plaque stabilization. However, the net beneficial impact of statins on VC and AVC is still debated [99,100], since it interferes with AVC [101] by promoting a deleterious effect on aortic valves [102]. Statins also interfere with the anti-calcifying mechanisms affecting SMC proliferation [103], and induce disturbances in the regulation of the extracellular nucleotidic pathways [104], or inhibition of vitamin K dependent factors [105]. Due to the direct role of PCSK9 on VC [94], anti-PCSK9 are expected to reduce this effect. The calcification effect of statins was attenuated when associated with anti-PCSK9 agents [106]. Addition of ezetimibe to statin therapy could also reduce plaque and lipid burdens, but may not modify plaque composition. Although current evidence supports a similar impact from the addition of PCSK9 inhibitors to statin therapy, more studies are needed to confirm such an effect [107]. Therefore, the debate about the opportunistic presence of calcification in the vascular wall or the aortic valves is far from being closed, and much is expected from the emerging non-lipid therapeutics targeting the inflammasome.

## 5. Conclusions

**Figure 3:**
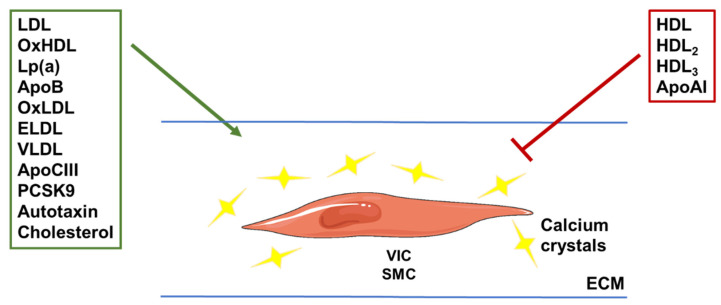
Illustration of the roles of different (apo)lipoproteins in vascular calcification. VLDL and LDL lipoprotein subfractions, and their oxidized or enzyme-modified forms (OxLDL and ELDL), were shown to stimulate calcium crystal deposition. Components of these particles, such as apoCIII, apoB and cholesterol, were also linked to increased calcification. The same was shown for Lp(a) and the enzyme autotaxin carried by these particles. The hepatic enzyme PCSK9 was shown to stimulate vascular calcification by increasing LDL concentrations through degradation of the LDL receptor. HDL subfractions and the main apolipoprotein found in these particles (apoAI) were shown to reduce calcification, although the oxidized form of HDL (OxHDL) was shown to do the opposite. VIC: valvular interstitial cell; SMC: smooth muscle cell; ECM: extra-cellular matrix.

VC, particularly those affecting aortic valves, represents a chronic disorder with increasing incidence worldwide. The link between lipoproteins and their related factors and VC (summarized in Figure 3) appears logical since inappropriate calcium deposition mainly occurs in tissues where the presence of lipids and the control of their metabolism is of extreme importance.

VC lacks specific pharmacological therapies. In particular, LDL lowering strategies (namely statins) have failed in clinical trials. It is thus of crucial importance to identify novel molecules that can be targeted to develop new therapeutic strategies. 

Moreover, clinicians are looking for more discriminating factors to better evaluate patients at risk of cardiovascular disease, particularly VC at the early stage, than those currently used, in order to propose personalized care. The relationship between calcification and lipoprotein sub-fractions could represent a promising avenue of this type of research. Indeed, specific pharmacological approaches treating lipoproteins with an effect on calcification can represent a novel venue for the treatment of this pathology. However, the role of lipoproteins and apolipoprotein remains insufficiently elucidated. In particular, there is an urgent need to identify the specific role of different lipoprotein components and related factors, also by using pre-clinical models. As an example, PCSK9 inhibitors led to a reduction of both LDL cholesterol and Lp(a). It is thus difficult to identify the specific factors controlling calcification. Thus, drugs based on PCSK9 and/or Lp(a) inhibition can represent promising molecules for the treatment of VC.

## References

[1] Song P., Fang Z., Wang H., Cai Y., Rahimi K., Zhu Y., Fowkes F.G.R., Fowkes F.J.I., Rudan I. (2020). Global and regional prevalence, burden, and risk factors for carotid atherosclerosis: A systematic review, meta-analysis, and modelling study. Lancet Glob. Health.

[2] Roth G.A., Mensah G.A., Johnson C.O., Addolorato G., Ammirati E., Baddour L.M., Barengo N.C., Beaton A.Z., Benjamin E.J., Benziger C.P. (2020). Global Burden of Cardiovascular Diseases and Risk Factors, 1990–2019: Update From the GBD 2019 Study. J. Am. Coll. Cardiol..

[3] Lusis A.J. (2000). Atherosclerosis. Nature.

[4] Libby P. (2002). Inflammation in atherosclerosis. Nature.

[5] Libby P., Aikawa M., Schonbeck U. (2000). Cholesterol and atherosclerosis. Biochim. Biophys. Acta.

[6] Shankman L.S., Gomez D., Cherepanova O.A., Salmon M., Alencar G.F., Haskins R.M., Swiatlowska P., Newman A.A., Greene E.S., Straub A.C. (2015). KLF4-dependent phenotypic modulation of smooth muscle cells has a key role in atherosclerotic plaque pathogenesis. Nat. Med..

[7] Robbins C.S., Hilgendorf I., Weber G.F., Theurl I., Iwamoto Y., Figueiredo J.L., Gorbatov R., Sukhova G.K., Gerhardt L.M., Smyth D. (2013). Local proliferation dominates lesional macrophage accumulation in atherosclerosis. Nat. Med..

[8] Tabas I. (2005). Consequences and therapeutic implications of macrophage apoptosis in atherosclerosis: The importance of lesion stage and phagocytic efficiency. Arterioscler. Thromb. Vasc. Biol..

[9] Tabas I. (2010). Macrophage death and defective inflammation resolution in atherosclerosis. Nat. Rev. Immunol..

[10] Libby P., Buring J.E., Badimon L., Hansson G.K., Deanfield J., Bittencourt M.S., Tokgozoglu L., Lewis E.F. (2019). Atherosclerosis. Nat. Rev. Dis. Prim..

[11] Gebbers J.O. (2007). Atherosclerosis, cholesterol, nutrition, and statins—A critical review. Ger. Med. Sci..

[12] Jakubiak G.K., Pawlas N., Cieslar G., Stanek A. (2021). Pathogenesis and Clinical Significance of In-Stent Restenosis in Patients with Diabetes. Int. J. Environ. Res. Public Health.

[13] Demer L.L., Tintut Y. (2008). Vascular calcification: Pathobiology of a multifaceted disease. Circulation.

[14] Sutton N.R., Malhotra R., St Hilaire C., Aikawa E., Blumenthal R.S., Gackenbach G., Goyal P., Johnson A., Nigwekar S.U., Shanahan C.M. (2023). Molecular Mechanisms of Vascular Health: Insights From Vascular Aging and Calcification. Arterioscler. Thromb. Vasc. Biol..

[15] Vieceli Dalla Sega F., Fortini F., Severi P., Rizzo P., Gardi I., Cimaglia P., Rapezzi C., Tavazzi L., Ferrari R. (2022). Cardiac Calcifications: Phenotypes, Mechanisms, Clinical and Prognostic Implications. Biology.

[16] Dos Santos V.P., Pozzan G., Castelli V., Caffaro R.A. (2021). Arteriosclerosis, atherosclerosis, arteriolosclerosis, and Monckeberg medial calcific sclerosis: What is the difference?. J. Vasc. Bras..

[17] Lee H.Y., Lim S., Park S. (2021). Role of Inflammation in Arterial Calcification. Korean Circ. J..

[18] St Hilaire C. (2022). Medial Arterial Calcification: A Significant and Independent Contributor of Peripheral Artery Disease. Arterioscler. Thromb. Vasc. Biol..

[19] Lanzer P., Boehm M., Sorribas V., Thiriet M., Janzen J., Zeller T., St Hilaire C., Shanahan C. (2014). Medial vascular calcification revisited: Review and perspectives. Eur. Heart J..

[20] Aboyans V., Lacroix P., Tran M.H., Salamagne C., Galinat S., Archambeaud F., Criqui M.H., Laskar M. (2011). The prognosis of diabetic patients with high ankle-brachial index depends on the coexistence of occlusive peripheral artery disease. J. Vasc. Surg..

[21] Chen N.X., Moe S.M. (2012). Vascular calcification: Pathophysiology and risk factors. Curr. Hypertens. Rep..

[22] Pan W., Jie W., Huang H. (2023). Vascular calcification: Molecular mechanisms and therapeutic interventions. MedComm.

[23] Rutsch F., Buers I., Nitschke Y. (2021). Hereditary Disorders of Cardiovascular Calcification. Arterioscler. Thromb. Vasc. Biol..

[24] Montanaro M., Scimeca M., Anemona L., Servadei F., Giacobbi E., Bonfiglio R., Bonanno E., Urbano N., Ippoliti A., Santeusanio G. (2021). The Paradox Effect of Calcification in Carotid Atherosclerosis: Microcalcification is Correlated with Plaque Instability. Int. J. Mol. Sci..

[25] Kelly-Arnold A., Maldonado N., Laudier D., Aikawa E., Cardoso L., Weinbaum S. (2013). Revised microcalcification hypothesis for fibrous cap rupture in human coronary arteries. Proc. Natl. Acad. Sci. USA.

[26] Jaminon A., Reesink K., Kroon A., Schurgers L. (2019). The Role of Vascular Smooth Muscle Cells in Arterial Remodeling: Focus on Calcification-Related Processes. Int. J. Mol. Sci..

[27] Abbasian N. (2021). Vascular Calcification Mechanisms: Updates and Renewed Insight into Signaling Pathways Involved in High Phosphate-Mediated Vascular Smooth Muscle Cell Calcification. Biomedicines.

[28] Waring O.J., Skenteris N.T., Biessen E.A.L., Donners M. (2022). Two-faced Janus: The dual role of macrophages in atherosclerotic calcification. Cardiovasc. Res..

[29] Li Y., Sun Z., Zhang L., Yan J., Shao C., Jing L., Li L., Wang Z. (2020). Role of Macrophages in the Progression and Regression of Vascular Calcification. Front. Pharmacol..

[30] Maddaloni E., Xia Y., Park K., D’Eon S., Tinsley L.J., St-Louis R., Khamaisi M., Li Q., King G.L., Keenan H.A. (2017). High density lipoprotein modulates osteocalcin expression in circulating monocytes: A potential protective mechanism for cardiovascular disease in type 1 diabetes. Cardiovasc. Diabetol..

[31] Mackey R.H., Kuller L.H., Sutton-Tyrrell K., Evans R.W., Holubkov R., Matthews K.A. (2002). Lipoprotein subclasses and coronary artery calcium in postmenopausal women from the healthy women study. Am. J. Cardiol..

[32] Yao T., Long Q., Li J., Li G., Ding Y., Cui Q., Liu Z. (2020). Small dense low-density lipoprotein cholesterol is strongly associated with NIHSS score and intracranial arterial calcification in acute ischemic stroke subjects. Sci. Rep..

[33] Cromwell W.C., Otvos J.D., Keyes M.J., Pencina M.J., Sullivan L., Vasan R.S., Wilson P.W., D’Agostino R.B. (2007). LDL Particle Number and Risk of Future Cardiovascular Disease in the Framingham Offspring Study—Implications for LDL Management. J. Clin. Lipidol..

[34] Prado K.B., Shugg S., Backstrand J.R. (2011). Low-density lipoprotein particle number predicts coronary artery calcification in asymptomatic adults at intermediate risk of cardiovascular disease. J. Clin. Lipidol..

[35] Zaid M., Miura K., Fujiyoshi A., Abbott R.D., Hisamatsu T., Kadota A., Arima H., Kadowaki S., Torii S., Miyagawa N. (2016). Associations of serum LDL particle concentration with carotid intima-media thickness and coronary artery calcification. J. Clin. Lipidol..

[36] Cao J., Nomura S.O., Steffen B.T., Guan W., Remaley A.T., Karger A.B., Ouyang P., Michos E.D., Tsai M.Y. (2020). Apolipoprotein B discordance with low-density lipoprotein cholesterol and non-high-density lipoprotein cholesterol in relation to coronary artery calcification in the Multi-Ethnic Study of Atherosclerosis (MESA). J. Clin. Lipidol..

[37] Chang T.Y., Chen J.D. (2021). Low-density lipoprotein cholesterol/apolipoprotein B ratio is superior to apolipoprotein B alone in the diagnosis of coronary artery calcification. Coron. Artery Dis..

[38] Sandesara P.B., Mehta A., O’Neal W.T., Mohamed Kelli H., Sathiyakumar V., Martin S.S., Blaha M.J., Blumenthal R.S., Sperling L.S. (2020). Association of Elevated High-Density Lipoprotein Cholesterol and Particle Concentration With Coronary Artery Calcium: The Multi-Ethnic Study of Atherosclerosis. Circ. Cardiovasc. Imaging.

[39] Mackey R.H., Greenland P., Goff D.C., Lloyd-Jones D., Sibley C.T., Mora S. (2012). High-density lipoprotein cholesterol and particle concentrations, carotid atherosclerosis, and coronary events: MESA (multi-ethnic study of atherosclerosis). J. Am. Coll. Cardiol..

[40] Voros S., Joshi P., Qian Z., Rinehart S., Vazquez-Figueroa J.G., Anderson H., Elashoff M., Murrieta L., Karmpaliotis D., Kalynych A. (2013). Apoprotein B, small-dense LDL and impaired HDL remodeling is associated with larger plaque burden and more noncalcified plaque as assessed by coronary CT angiography and intravascular ultrasound with radiofrequency backscatter: Results from the ATLANTA I study. J. Am. Heart Assoc..

[41] Armstrong M.K., Fraser B.J., Hartiala O., Buscot M.J., Juonala M., Wu F., Koskinen J., Hutri-Kahonen N., Kahonen M., Laitinen T.P. (2021). Association of Non-High-Density Lipoprotein Cholesterol Measured in Adolescence, Young Adulthood, and Mid-Adulthood With Coronary Artery Calcification Measured in Mid-Adulthood. JAMA Cardiol..

[42] Edwards M.K., Blaha M.J., Loprinzi P.D. (2017). Atherogenic Index of Plasma and Triglyceride/High-Density Lipoprotein Cholesterol Ratio Predict Mortality Risk Better Than Individual Cholesterol Risk Factors, Among an Older Adult Population. Mayo Clin. Proc..

[43] Won K.B., Han D., Lee J.H., Choi S.Y., Chun E.J., Park S.H., Han H.W., Sung J., Jung H.O., Chang H.J. (2020). Atherogenic index of plasma and coronary artery calcification progression beyond traditional risk factors according to baseline coronary artery calcium score. Sci. Rep..

[44] Lommi J.I., Kovanen P.T., Jauhiainen M., Lee-Rueckert M., Kupari M., Helske S. (2011). High-density lipoproteins (HDL) are present in stenotic aortic valves and may interfere with the mechanisms of valvular calcification. Atherosclerosis.

[45] Torzewski M., Ravandi A., Yeang C., Edel A., Bhindi R., Kath S., Twardowski L., Schmid J., Yang X., Franke U.F.W. (2017). Lipoprotein(a) Associated Molecules are Prominent Components in Plasma and Valve Leaflets in Calcific Aortic Valve Stenosis. JACC Basic Transl. Sci..

[46] Schlotter F., de Freitas R.C.C., Rogers M.A., Blaser M.C., Wu P.J., Higashi H., Halu A., Iqbal F., Andraski A.B., Rodia C.N. (2021). ApoC-III is a novel inducer of calcification in human aortic valves. J. Biol. Chem..

[47] Gerber Y., Goldbourt U., Feinberg M.S., Segev S., Harats D. (2003). Are triglyceride-rich lipoproteins associated with aortic valve sclerosis? A preliminary report. Atherosclerosis.

[48] Pollin T.I., Damcott C.M., Shen H., Ott S.H., Shelton J., Horenstein R.B., Post W., McLenithan J.C., Bielak L.F., Peyser P.A. (2008). A null mutation in human APOC3 confers a favorable plasma lipid profile and apparent cardioprotection. Science.

[49] Capoulade R., Torzewski M., Mayr M., Chan K.L., Mathieu P., Bosse Y., Dumesnil J.G., Tam J., Teo K.K., Burnap S.A. (2020). ApoCIII-Lp(a) complexes in conjunction with Lp(a)-OxPL predict rapid progression of aortic stenosis. Heart.

[50] Qiao Y.N., Zou Y.L., Guo S.D. (2022). Low-density lipoprotein particles in atherosclerosis. Front. Physiol..

[51] Sun J.T., Chen Y.Y., Mao J.Y., Wang Y.P., Chen Y.F., Hu X., Yang K., Liu Y. (2019). Oxidized HDL, as a Novel Biomarker for Calcific Aortic Valve Disease, Promotes the Calcification of Aortic Valve Interstitial Cells. J. Cardiovasc. Transl. Res..

[52] Miki T., Miyoshi T., Kotani K., Kohno K., Asonuma H., Sakuragi S., Koyama Y., Nakamura K., Ito H. (2019). Decrease in oxidized high-density lipoprotein is associated with slowed progression of coronary artery calcification: Subanalysis of a prospective multicenter study. Atherosclerosis.

[53] Kakino A., Usami Y., Horiuchi S., Fujita Y., Kotani K., Chen C.H., Okamura T., Sawamura T. (2019). A Novel Cell-Free, Non-Fluorescent Method to Measure LOX-1-Binding Activity Corresponding to The Functional Activity of HDL. J. Atheroscler. Thromb..

[54] Hirata A., Kakino A., Okamura T., Usami Y., Fujita Y., Kadota A., Fujiyoshi A., Hisamatsu T., Kondo K., Segawa H. (2020). The relationship between serum levels of LOX-1 ligand containing ApoAI as a novel marker of dysfunctional HDL and coronary artery calcification in middle-aged Japanese men. Atherosclerosis.

[55] Ten Kate G.R., Bos S., Dedic A., Neefjes L.A., Kurata A., Langendonk J.G., Liem A., Moelker A., Krestin G.P., de Feyter P.J. (2015). Increased Aortic Valve Calcification in Familial Hypercholesterolemia: Prevalence, Extent, and Associated Risk Factors. J. Am. Coll. Cardiol..

[56] Al Kindi M., Belanger A.M., Sayegh K., Senouci S., Aljenedil S., Sivakumaran L., Ruel I., Al Rasadi K., Al Waili K., Awan Z. (2017). Aortic Calcification Progression in Heterozygote Familial Hypercholesterolemia. Can. J. Cardiol..

[57] Koschinsky M.L., Marcovina S.M. (2004). Structure-function relationships in apolipoprotein(a): Insights into lipoprotein(a) assembly and pathogenicity. Curr. Opin. Lipidol..

[58] Duarte Lau F., Giugliano R.P. (2022). Lipoprotein(a) and its Significance in Cardiovascular Disease: A Review. JAMA Cardiol.

[59] Tsimikas S. (2017). A Test in Context: Lipoprotein(a): Diagnosis, Prognosis, Controversies, and Emerging Therapies. J. Am. Coll. Cardiol..

[60] Tsimikas S. (2019). Potential Causality and Emerging Medical Therapies for Lipoprotein(a) and Its Associated Oxidized Phospholipids in Calcific Aortic Valve Stenosis. Circ. Res..

[61] Bouchareb R., Mahmut A., Nsaibia M.J., Boulanger M.C., Dahou A., Lepine J.L., Laflamme M.H., Hadji F., Couture C., Trahan S. (2015). Autotaxin Derived From Lipoprotein(a) and Valve Interstitial Cells Promotes Inflammation and Mineralization of the Aortic Valve. Circulation.

[62] Peng J., Liu M.M., Liu H.H., Xu R.X., Zhu C.G., Guo Y.L., Wu N.Q., Dong Q., Cui C.J., Li J.J. (2022). Lipoprotein (a)-mediated vascular calcification: Population-based and in vitro studies. Metabolism.

[63] Zheng K.H., Tsimikas S., Pawade T., Kroon J., Jenkins W.S.A., Doris M.K., White A.C., Timmers N., Hjortnaes J., Rogers M.A. (2019). Lipoprotein(a) and Oxidized Phospholipids Promote Valve Calcification in Patients With Aortic Stenosis. J. Am. Coll. Cardiol..

[64] Despres A.A., Perrot N., Poulin A., Tastet L., Shen M., Chen H.Y., Bourgeois R., Trottier M., Tessier M., Guimond J. (2019). Lipoprotein(a), Oxidized Phospholipids, and Aortic Valve Microcalcification Assessed by 18F-Sodium Fluoride Positron Emission Tomography and Computed Tomography. CJC Open.

[65] Pechlivanis S., Mahabadi A.A., Hoffmann P., Nothen M.M., Broecker-Preuss M., Erbel R., Moebus S., Stang A., Jockel K.H. (2020). Association between lipoprotein(a) (Lp(a)) levels and Lp(a) genetic variants with coronary artery calcification. BMC Med. Genet..

[66] Arsenault B.J., Boekholdt S.M., Dube M.P., Rheaume E., Wareham N.J., Khaw K.T., Sandhu M.S., Tardif J.C. (2014). Lipoprotein(a) levels, genotype, and incident aortic valve stenosis: A prospective Mendelian randomization study and replication in a case-control cohort. Circ. Cardiovasc. Genet..

[67] Bergmark B.A., O’Donoghue M.L., Murphy S.A., Kuder J.F., Ezhov M.V., Ceska R., Gouni-Berthold I., Jensen H.K., Tokgozoglu S.L., Mach F. (2020). An Exploratory Analysis of Proprotein Convertase Subtilisin/Kexin Type 9 Inhibition and Aortic Stenosis in the FOURIER Trial. JAMA Cardiol..

[68] Wodaje T., Littmann K., Habel H., Bottai M., Back M., Parini P., Brinck J. (2022). Plasma Lipoprotein(a) measured in routine clinical care and the association with incident calcified aortic valve stenosis during a 14-year observational period. Atherosclerosis.

[69] Vongpromek R., Bos S., Ten Kate G.J., Yahya R., Verhoeven A.J., de Feyter P.J., Kronenberg F., Roeters van Lennep J.E., Sijbrands E.J., Mulder M.T. (2015). Lipoprotein(a) levels are associated with aortic valve calcification in asymptomatic patients with familial hypercholesterolaemia. J. Intern. Med..

[70] Kaiser Y., Singh S.S., Zheng K.H., Verbeek R., Kavousi M., Pinto S.J., Vernooij M.W., Sijbrands E.J.G., Boekholdt S.M., de Rijke Y.B. (2021). Lipoprotein(a) is robustly associated with aortic valve calcium. Heart.

[71] Kaiser Y., van der Toorn J.E., Singh S.S., Zheng K.H., Kavousi M., Sijbrands E.J.G., Stroes E.S.G., Vernooij M.W., de Rijke Y.B., Boekholdt S.M. (2022). Lipoprotein(a) is associated with the onset but not the progression of aortic valve calcification. Eur. Heart J..

[72] Capoulade R., Chan K.L., Yeang C., Mathieu P., Bosse Y., Dumesnil J.G., Tam J.W., Teo K.K., Mahmut A., Yang X. (2015). Oxidized Phospholipids, Lipoprotein(a), and Progression of Calcific Aortic Valve Stenosis. J. Am. Coll. Cardiol..

[73] Nsaibia M.J., Mahmut A., Boulanger M.C., Arsenault B.J., Bouchareb R., Simard S., Witztum J.L., Clavel M.A., Pibarot P., Bosse Y. (2016). Autotaxin interacts with lipoprotein(a) and oxidized phospholipids in predicting the risk of calcific aortic valve stenosis in patients with coronary artery disease. J. Intern. Med..

[74] Poggio P., Songia P., Cavallotti L., Barbieri S.S., Zanotti I., Arsenault B.J., Valerio V., Ferri N., Capoulade R., Camera M. (2018). PCSK9 Involvement in Aortic Valve Calcification. J. Am. Coll. Cardiol..

[75] Perrot N., Valerio V., Moschetta D., Boekholdt S.M., Dina C., Chen H.Y., Abner E., Martinsson A., Manikpurage H.D., Rigade S. (2020). Genetic and In Vitro Inhibition of PCSK9 and Calcific Aortic Valve Stenosis. JACC Basic Transl. Sci..

[76] Langsted A., Nordestgaard B.G., Benn M., Tybjaerg-Hansen A., Kamstrup P.R. (2016). PCSK9 R46L Loss-of-Function Mutation Reduces Lipoprotein(a), LDL Cholesterol, and Risk of Aortic Valve Stenosis. J. Clin. Endocrinol. Metab..

[77] Gao F., Li Y.P., Ma X.T., Wang Z.J., Shi D.M., Zhou Y.J. (2022). Effect of Alirocumab on Coronary Calcification in Patients With Coronary Artery Disease. Front. Cardiovasc. Med..

[78] Goettsch C., Strzelecka-Kiliszek A., Bessueille L., Quillard T., Mechtouff L., Pikula S., Canet-Soulas E., Millan J.L., Fonta C., Magne D. (2022). TNAP as a therapeutic target for cardiovascular calcification: A discussion of its pleiotropic functions in the body. Cardiovasc. Res..

[79] Parhami F., Basseri B., Hwang J., Tintut Y., Demer L.L. (2002). High-density lipoprotein regulates calcification of vascular cells. Circ. Res..

[80] Harun N.H., Froemming G.R.A., Nawawi H.M., Muid S.A. (2019). Inflammation and Vascular Calcification Causing Effects of Oxidized HDL are Attenuated by Adiponectin in Human Vascular Smooth Muscle Cells. Int. J. Mol. Cell. Med..

[81] Twardowski L., Cheng F., Michaelsen J., Winter S., Hofmann U., Schaeffeler E., Muller S., Sonnenberg M., Steuer K., Ott G. (2015). Enzymatically Modified Low-Density Lipoprotein Is Present in All Stages of Aortic Valve Sclerosis: Implications for Pathogenesis of the Disease. J. Am. Heart Assoc..

[82] Chellan B., Rojas E., Zhang C., Hofmann Bowman M.A. (2018). Enzyme-modified non-oxidized LDL (ELDL) induces human coronary artery smooth muscle cell transformation to a migratory and osteoblast-like phenotype. Sci. Rep..

[83] Nadlonek N.A., Lee J.H., Weyant M.J., Meng X., Fullerton D.A. (2013). ox-LDL induces PiT-1 expression in human aortic valve interstitial cells. J. Surg. Res..

[84] Maziere C., Salle V., Gomila C., Maziere J.C. (2013). Oxidized low density lipoprotein increases RANKL level in human vascular cells. Involvement of oxidative stress. Biochem. Biophys. Res. Commun..

[85] Nsaibia M.J., Boulanger M.C., Bouchareb R., Mkannez G., Le Quang K., Hadji F., Argaud D., Dahou A., Bosse Y., Koschinsky M.L. (2017). OxLDL-derived lysophosphatidic acid promotes the progression of aortic valve stenosis through a LPAR1-RhoA-NF-kappaB pathway. Cardiovasc. Res..

[86] Geng Y., Hsu J.J., Lu J., Ting T.C., Miyazaki M., Demer L.L., Tintut Y. (2011). Role of cellular cholesterol metabolism in vascular cell calcification. J. Biol. Chem..

[87] Watson K.E., Bostrom K., Ravindranath R., Lam T., Norton B., Demer L.L. (1994). TGF-beta 1 and 25-hydroxycholesterol stimulate osteoblast-like vascular cells to calcify. J. Clin. Investig..

[88] Schutkowski A., Hirche F., Geissler S., Radtke J., Stangl G.I. (2015). Additive effects of lupin protein and phytic acid on aortic calcification in ApoE deficient mice. J. Clin. Transl. Endocrinol..

[89] Speidl W.S., Cimmino G., Ibanez B., Elmariah S., Hutter R., Garcia M.J., Fuster V., Goldman M.E., Badimon J.J. (2010). Recombinant apolipoprotein A-I Milano rapidly reverses aortic valve stenosis and decreases leaflet inflammation in an experimental rabbit model. Eur. Heart J..

[90] Trapeaux J., Busseuil D., Shi Y., Nobari S., Shustik D., Mecteau M., El-Hamamsy I., Lebel M., Mongrain R., Rheaume E. (2013). Improvement of aortic valve stenosis by ApoA-I mimetic therapy is associated with decreased aortic root and valve remodelling in mice. Br. J. Pharmacol..

[91] Busseuil D., Shi Y., Mecteau M., Brand G., Kernaleguen A.E., Thorin E., Latour J.G., Rheaume E., Tardif J.C. (2008). Regression of aortic valve stenosis by ApoA-I mimetic peptide infusions in rabbits. Br. J. Pharmacol..

[92] Rogers M.A., Atkins S.K., Zheng K.H., Singh S.A., Chelvanambi S., Pham T.H., Kuraoka S., Stroes E.S.G., Aikawa M., Aikawa E. (2022). Lipoprotein(a) Induces Vesicular Cardiovascular Calcification Revealed With Single-Extracellular Vesicle Analysis. Front. Cardiovasc. Med..

[93] Que X., Hung M.Y., Yeang C., Gonen A., Prohaska T.A., Sun X., Diehl C., Maatta A., Gaddis D.E., Bowden K. (2018). Oxidized phospholipids are proinflammatory and proatherogenic in hypercholesterolaemic mice. Nature.

[94] Lupo M.G., Bressan A., Donato M., Canzano P., Camera M., Poggio P., Greco M.F., Garofalo M., De Martin S., Panighel G. (2022). PCSK9 promotes arterial medial calcification. Atherosclerosis.

[95] Lee S.E., Chang H.J., Sung J.M., Park H.B., Heo R., Rizvi A., Lin F.Y., Kumar A., Hadamitzky M., Kim Y.J. (2018). Effects of Statins on Coronary Atherosclerotic Plaques: The PARADIGM Study. JACC Cardiovasc. Imaging.

[96] Lee S.H., Choi J.H. (2018). Involvement of inflammatory responses in the early development of calcific aortic valve disease: Lessons from statin therapy. Anim. Cells Syst..

[97] Chen Z., Qureshi A.R., Parini P., Hurt-Camejo E., Ripsweden J., Brismar T.B., Barany P., Jaminon A.M., Schurgers L.J., Heimburger O. (2017). Does statins promote vascular calcification in chronic kidney disease?. Eur. J. Clin. Investig..

[98] Saremi A., Bahn G., Reaven P.D., Investigators V. (2012). Progression of vascular calcification is increased with statin use in the Veterans Affairs Diabetes Trial (VADT). Diabetes Care.

[99] Hermans H., Herijgers P., Holvoet P., Verbeken E., Meuris B., Flameng W., Herregods M.C. (2010). Statins for calcific aortic valve stenosis: Into oblivion after SALTIRE and SEAS? An extensive review from bench to bedside. Curr. Probl. Cardiol..

[100] Zhao Y., Nicoll R., He Y.H., Henein M.Y. (2016). The effect of statins on valve function and calcification in aortic stenosis: A meta-analysis. Atherosclerosis.

[101] Puri R., Nicholls S.J., Shao M., Kataoka Y., Uno K., Kapadia S.R., Tuzcu E.M., Nissen S.E. (2015). Impact of statins on serial coronary calcification during atheroma progression and regression. J. Am. Coll. Cardiol..

[102] Mazzone A., Clemente A., Chiappino D., Berti S., Vassalle C. (2018). Double Face of Statins at the Crossroad of Coronary Atherosclerotic Plaque and Aortic Valve Calcification?. JACC Cardiovasc. Imaging.

[103] Trion A., Schutte-Bart C., Bax W.H., Jukema J.W., van der Laarse A. (2008). Modulation of calcification of vascular smooth muscle cells in culture by calcium antagonists, statins, and their combination. Mol. Cell. Biochem..

[104] Mahmut A., Boulanger M.C., Bouchareb R., Hadji F., Mathieu P. (2015). Adenosine derived from ecto-nucleotidases in calcific aortic valve disease promotes mineralization through A2a adenosine receptor. Cardiovasc. Res..

[105] Zhelyazkova-Savova M.D., Yotov Y.T., Nikolova M.N., Nazifova-Tasinova N.F., Vankova D.G., Atanasov A.A., Galunska B.T. (2021). Statins, vascular calcification, and vitamin K-dependent proteins: Is there a relation?. Kaohsiung J. Med. Sci..

[106] Ikegami Y., Inoue I., Inoue K., Shinoda Y., Iida S., Goto S., Nakano T., Shimada A., Noda M. (2018). The annual rate of coronary artery calcification with combination therapy with a PCSK9 inhibitor and a statin is lower than that with statin monotherapy. NPJ Aging Mech. Dis..

[107] Liang D., Li C., Tu Y., Li Z., Zhang M. (2022). Additive effects of ezetimibe, evolocumab, and alirocumab on plaque burden and lipid content as assessed by intravascular ultrasound: A PRISMA-compliant meta-analysis. Medicine.

